# Binase treatment increases interferon sensitivity and apoptosis in SiHa cervical carcinoma cells by downregulating E6 and E7 human papilloma virus oncoproteins

**DOI:** 10.18632/oncotarget.20199

**Published:** 2017-08-10

**Authors:** Vladimir A. Mitkevich, Ksenia M. Burnysheva, Irina Yu Petrushanko, Alexei A. Adzhubei, Alexey A. Schulga, Peter M. Chumakov, Alexander A. Makarov

**Affiliations:** ^1^ Engelhardt Institute of Molecular Biology, Russian Academy of Sciences, 119991 Moscow, Russia; ^2^ Shemyakin-Ovchinnikov Institute of Bioorganic Chemistry, Russian Academy of Sciences, 117871 Moscow, Russia

**Keywords:** cytotoxic RNase, HPV, cancer cells, apoptosis, viral oncoproteins

## Abstract

In this study, we determined whether binase, a ribonuclease from *Bacillus pumilus*, increases interferon sensitivity and apoptosis in SiHa cervical cancer cells infected with high-risk human papilloma virus (HPV) strain 16. Binase treatment increased SiHa cell apoptosis in a time- and concentration-dependent manner, as determined by flow cytometry, WST tests and real time xCelligence cell index analysis. Binase-treated SiHa cells showed reduced expression of E6 and E7 viral oncoproteins and increased expression of their intracellular targets, p53 and pRb. Combined treatment with binase and IFNα2b enhanced the interferon sensitivity of HPV-positive SiHa cells. By contrast, combined treatment with binase and IFNα2b in HPV-negative C33A cervical cancer cells, which do no expess E6 and E7, elicited no changes in interferon sensitivity or p53 and pRb expression. These findings suggest binase enhances interferon sensitivity and apoptosis in HPV-positive SiHa cervical cancer cells by suppressing E6 and E7 viral protein expression.

## INTRODUCTION

Human papilloma virus (HPV) infection is one of the most common causes of sexually transmitted diseases worldwide. High-risk strains of the virus (HPV-16 and HPV-18) are linked to cancers of the cervix, anus, vulva, vagina, penis, mouth, head and neck in untreated individuals [1]. Majority of cervical cancer cases are HPV related, with 70% of cases infected with HPV-16 and HPV-18 [2]. More than 90 percent of anal cancers are caused by HPV, especially HPV-16 [3]. About 70 percent of oropharyngeal cancers are caused by HPV, with more than 50% cases linked to HPV-16 [4].

Mechanisms of malignant transformation of cells by HPV are well understood [5]. To promote virus replication in HPV infected epithelial cells, the E6 and E7 viral proteins interfere with cellular functions like cell division and stress surveillance. These changes result in uncontrolled divisions of the infected cells coupled to their escape from apoptosis. The E7 and E6 viral proteins target and inhibit the tumor suppressor proteins, pRb and p53, which results in malignant transformation [6]. In resting cells, the retinoblastoma protein (pRb) prevents unscheduled DNA replication by blocking the E2F transcription factor, which drives transcription of the S-phase genes [6]. The viral E7 protein binds to pRb, thereby releasing E2F. Unscheduled DNA replication elicits p53 pathway in normal cells, which results in apoptosis. To counteract this response, the viral E6 protein directs ubiquitin-mediated proteasome degradation of p53. As a result, HPV infected cells become resistant to p53-mediated apoptosis. Besides, HPV-specific proteins interfere with multiple components of the interferon (IFN) signaling pathway to circumvent immune surveillance [7]. IFN therapy is recommended to treat HPV infections since IFN signaling is necessary for anti-viral immunity [7]. Patients with genital warts induced by low-risk HPV types respond well to interferon treatment [5]. But, high-risk HPVs downregulate IFN, which renders IFN therapy ineffective [6, 7]. However, IFN therapy could be beneficial for HPV-positive cancers if combined with treatments that abrogate the virus-mediated effects and restore IFN signaling. However, treatments that specifically target HPV-mediated effects in cancer cells are currently not available.

Ribonucleases (RNases) are promising tools to cure neoplasia and viral infections [8]. They selectively eliminate various types of cancer cells [9, 10] and suppress replication of a number of viruses [11]. Binase is the RNase from *Bacillus pumilus*, which is an efficient therapeutic agent against various types of malignant cells [12, 13]. The toxicity of binase depends on the expression levels of *KIT*, *AML1-ETO, FLT*3 and *RAS* oncogenes in different cell systems [14–16]. For instance, binase treatment decreases KIT oncogenic protein expression, thereby killing Kasumi-1 acute myeloid leukemia cells [14]. Binase does not initiate necrosis in cell cultures and *in vivo* mouse tumor models, but inhibits tumor growth and metastasis [17]. Besides, binase treatment is not associated with toxicity in mice and promotes liver regeneration in mice bearing Lewis lung carcinoma cells [18]. Binase also demonstrates anti-viral activity against rabies, murrain and influenza viruses [11, 19]. Therefore, we investigated if binase could potentiate specific cytotoxic effects against SiHa cervical cancer cells that are transformed by the high risk HPV16 and restore interferon sensitivity in infected cells.

## RESULTS

### Binase treatment decreases viability of HPV-positive SiHa cells

**Figure 1:**
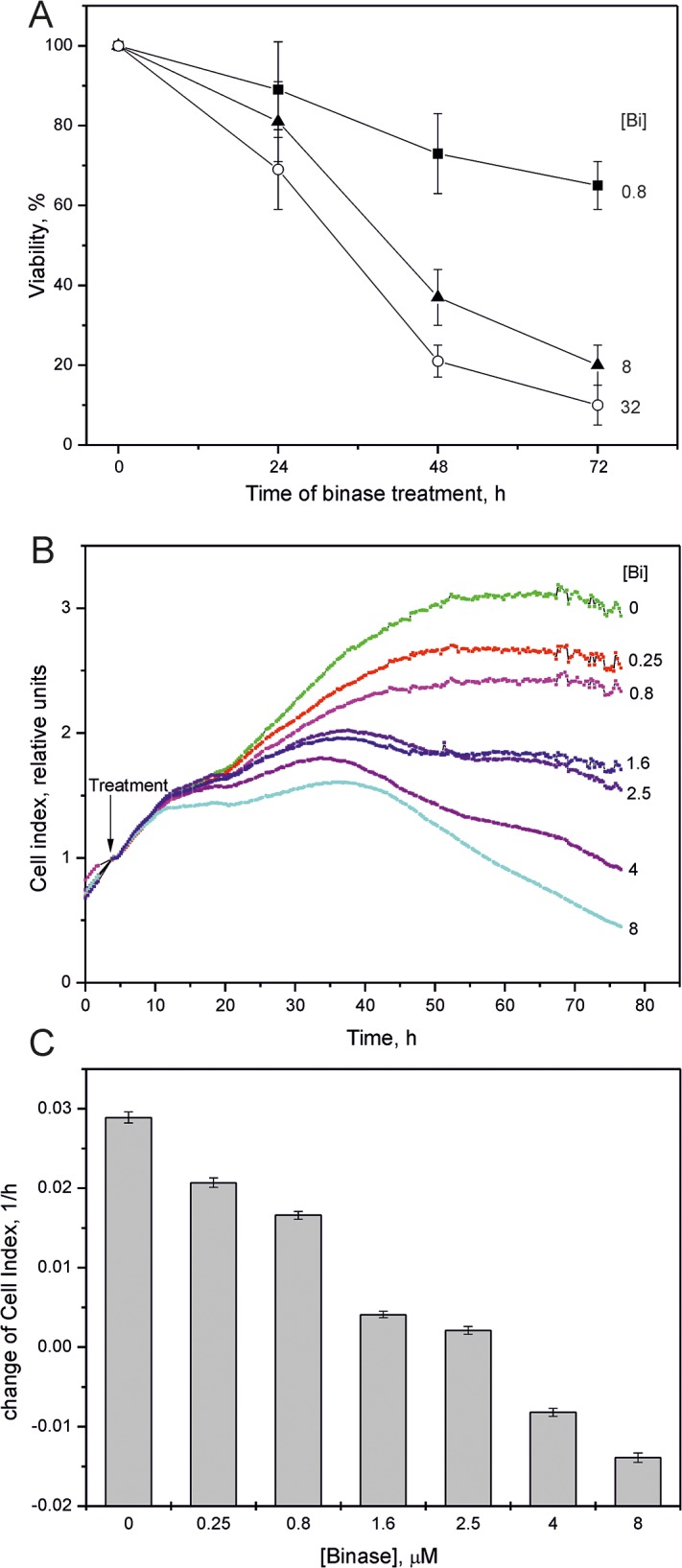
Effect of binase on SiHa cells **(A)** WST-1 assay showing percent viability of SiHa cells treated with 0.8, 8 and 32 μM binase for 24, 48 and 72 h relative to control without binase treatment. Data represents mean± SD of at least three independent experiments with triplicate samples each. **(B)** Real-time cell index changes in SiHa cells treated with or without 0.25-8 μM binase for 72 h using xCELLigence real time cell analyser. Arrow indicates the treatment time. Cells were grown in multiple wells of an E16-plate and the values represent average of three measurements. **(C)** The change in the rate of cell indexes of SiHa cells treated with or without binase in a 4-76 h time frame are shown.

**Figure 2:**
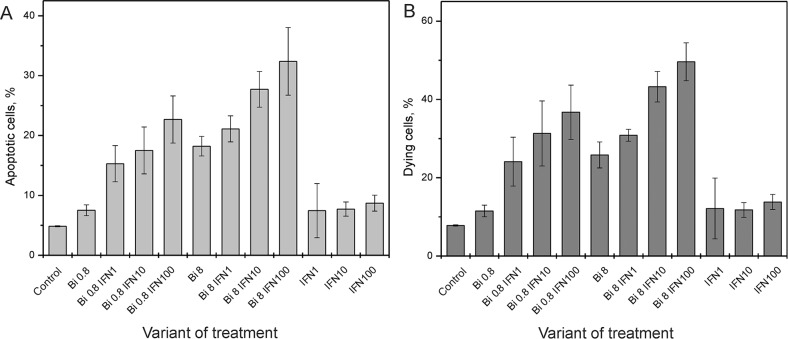
Effect of binase on SiHa cell death Flow cytometry analysis of **(A)** apoptotic and **(B)** dying HPV-positive SiHa cells treated with 0.8 and 8 μM binase with or without 1, 10 and 100 ng/ml INFα2b at 48 h. The AnnexinV^+^ PI^−^ cells were considered apoptotic (lower right quadrant), while AnnexinV^+^ PI^+^ (upper right quadrant), AnnexinV^+^ PI^−^ (lower right quadrant) and AnnexinV^−^ PI^+^ (upper left quadrant) were considered as dying cells. The values represent mean± SD of at least three independent experiments performed in triplicate.

To test the effects of binase on HPV-positive cervical carcinoma cells, we treated SiHa cells with different concentrations of binase. The viability of SiHa cells treated with 0.8, 8 and 32 μM binase decreased by 26, 63 and 79%, respectively at 48h and 35, 80 and 90%, respectively at 72 h (Figure 1A). The IC_50_ of binase at 72 h (concentration at 50% cell death) was 1.2±0.2 μM (Supplementary Figure 1). Cell viability correlated with dynamic changes in cell index based on xCELLigence real time cell analysis (Figure 1B, 1C). FACS analysis with AnnexinV/propidium iodide double staining showed that treatment with 0.8 and 8 μM binase for 48 h resulted in 7% and 18% apoptotic cells, respectively (Figure 2A) and 11.5% and 26% dying cells, respectively (Figure 2B). The fraction of necrotic cells was less than 1% for all samples treated with binase.

### Binase downregulates HPV E6 and E7 proteins in SiHa cells

**Figure 3:**
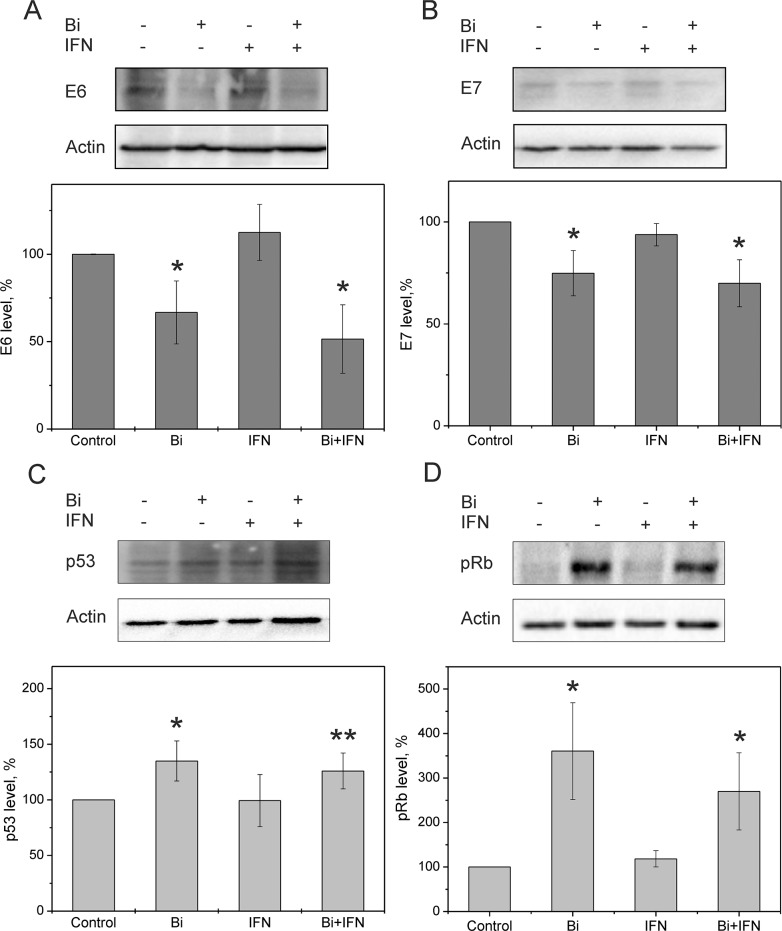
Expression of E6 and E7 HPV oncoproteins and their cellular targets, p53 and pRb in binase treated SiHa cells Representative western blot and bar graph shows **(A)** E6 **(B)** E7 **(C)** p53 and **(D)** pRb protein levels in SiHa cells treated with 8 μM binase with or without 100 ng/ml INFα2b for 48 h, relative to untreated control cells. Each bar represents mean±SD value from three independent experiments. Note: * denotes p <0.05; ** denotes p=0.07, compared to control.

We previously showed that the efficacy of binase against cancer cells was dependent on the expression levels of oncogenic proteins such as *cKIT, AML1-ETO, FLT3* and *RAS* [14–16]. Since the two HPV-16 oncogenes, *E6* and *E7* determine the transformation status of SiHa cells, we determined the effects of binase on the levels of E6 and E7 proteins and their host-cell targets p53 and pRb. Intracellular levels of E6 and E7 proteins were significantly decreased at 48 h after treating SiHa cells with 8 μM binase (Figure 3A, 3B). Concurrently, p53 and pRb levels increased by 1.5- and 3-fold, respectively (Figure 3C, 3D). These results demonstrated that binase downregulated E6 and E7 viral proteins, while up-regulating the p53 and pRb in SiHa cells.

### Binase treatment enhances interferon sensitivity of SiHa cells

**Figure 4:**
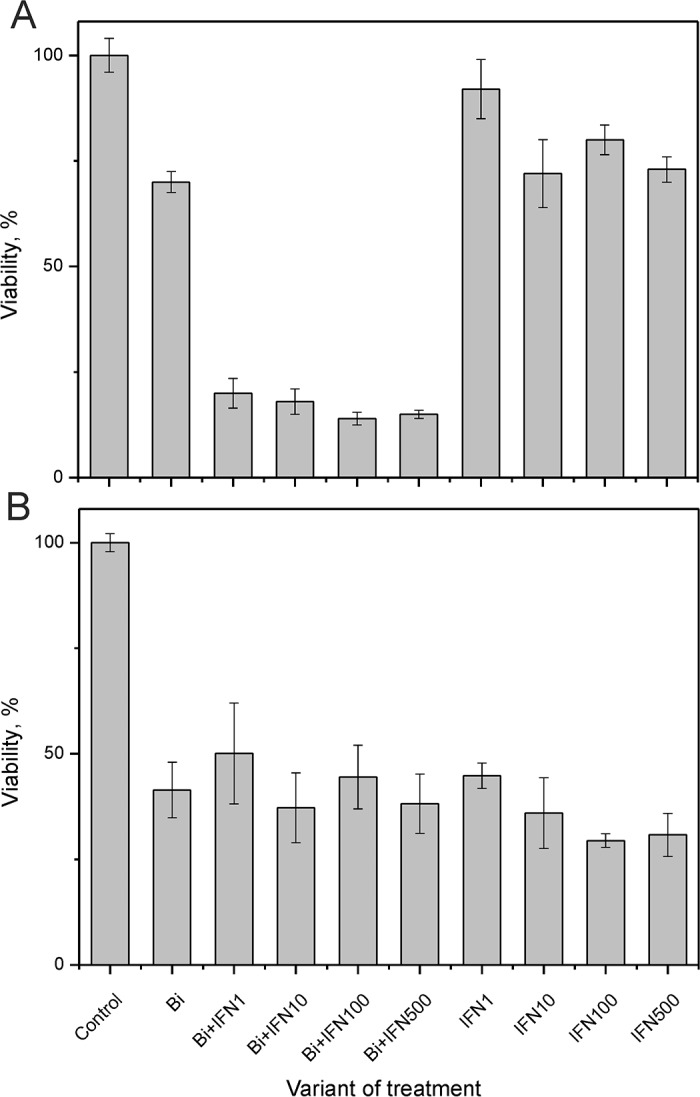
Effect of binase and INFα2b on viability of SiHa and C33A cells Percent viability of **(A)** SiHa and **(B)** C33A cells treated with 0.8 μM and 8 μM binase, respectively with or without 1, 10, 100 and 500 ng/ml INFα2b, relative to control without treatment at 72 h. Each value represents mean± SD of at least three independent experiments performed in triplicate.

**Figure 5:**
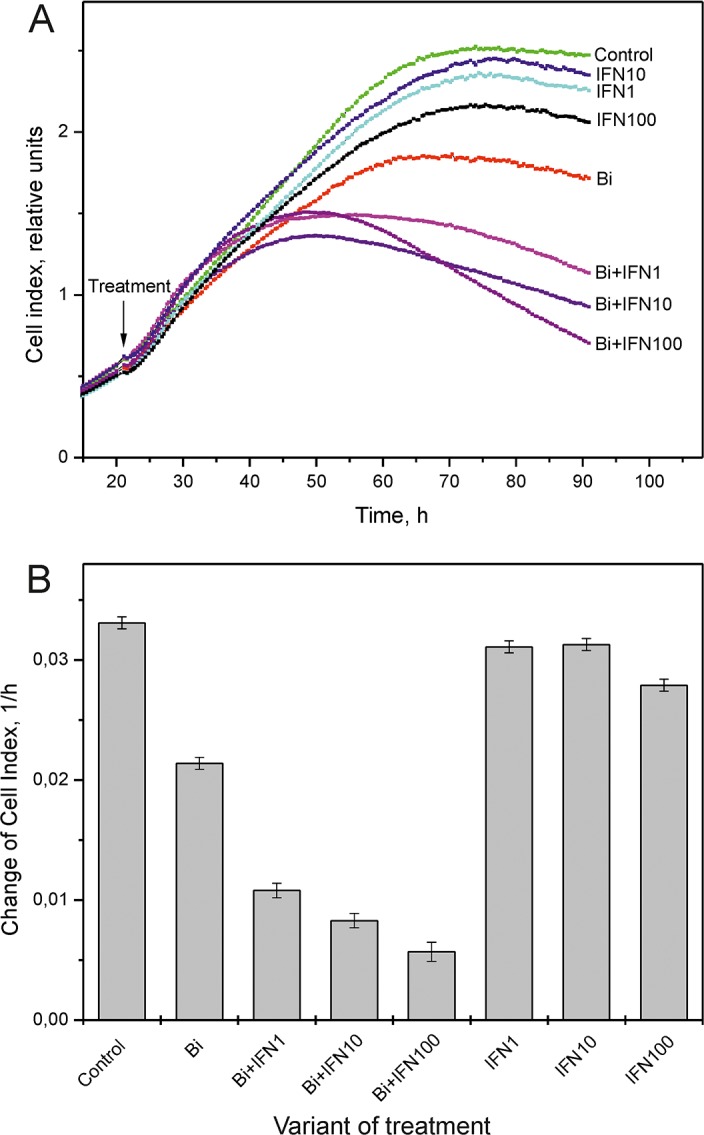
Real time cell index analysis of SiHa cells treated with binase and INFα2b combination **(A)** Real-time cell index analysis of SiHa cells treated with 0.8 μM binase with or without 1, 10 and 100 ng/ml INFα2b for 70 h. Arrow denotes treatment start time. Cells were grown in multiple wells of a E16 plate and the values represent average of three measurements. **(B)** The change of cell indexes of SiHa cells treated with 0.8 μM binase with or without 1, 10 and 100 ng/ml INFα2b between 21-91 h time points.

The viral E6 and E7 proteins render HPV-infected cells resistant to treatment with type I IFNs [20]. Since binase treatment suppressed E6 and E7 levels in SiHa cells, we investigated if binase treatment restored interferon response of HPV-positive cells. Incubation of SiHa cells with 1-500 ng/ml of IFNα2b alone for 72 h did not affect viability (Figure 4). But, combined treatment with 0.8 μM binase and 1-500 ng/ml IFNα2b reduced SiHa cell viability by 30-90% (Figure 4). Real time analysis of changes in the cell index showed a correlation between increased cell index changes and decreased viability in SiHa cells treated with a combination of binase and IFNα2b (Figure 5A, 5B). The toxicity of binase was evident at 24 h and progressed substantially during the next 24 h (Figure 5A). While treatment of SiHa cells with 1-100 ng/ml IFNα2b did not affect viability, the combined treatment of binase and IFNα2b induced significant apoptosis in a dose-dependent manner (Figure 2). Moreover, treatment with 100 ng/ml IFNα2b did not affect expression of E6, E7, p53 and pRb proteins at 48 h (Figure 3). But, combined treatment with 100 ng/ml IFNα2b and 8 μM of binase decreased E6 and E7 and increased p53 and pRb levels in SiHa cells similar to binase-only treatment (Figure 3).

### Binase does not increase interferon sensitivity of HPV-negative C33A cervical carcinoma cells

**Figure 6:**
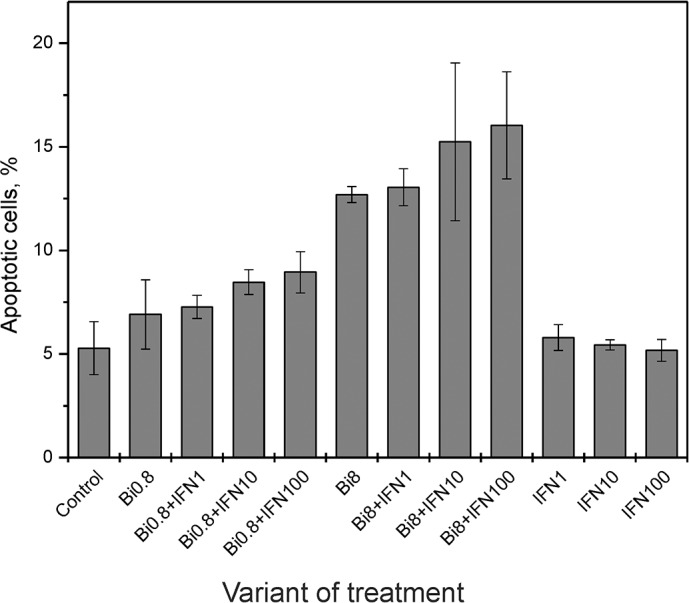
Effect of combined binase and INFα2b treatment on C33A cell death Flow cytometry analysis of percent apoptosis in HPV-negative C33A cells treated with 0.8 and 8 μM binase with or without 1, 10 and 100 ng/ml INFα2b (INF) at 48 h. Each value represents the mean± SD of at least three independent experiments performed in triplicate.

**Figure 7:**
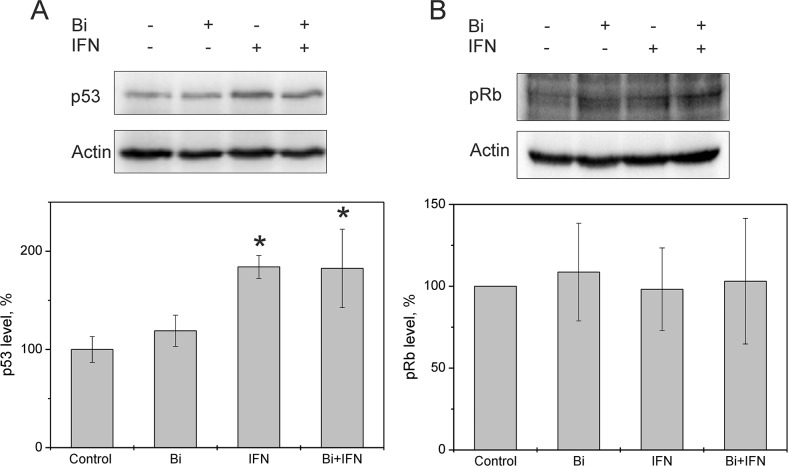
Effect of combined binase and INFα2b treatment on p53 and pRb levels in C33A cells Representative western blot and bar graph showing levels of **(A)** p53 and **(B)** pRb in C33A cells treated with 8 μM binase, with or without 100 ng/ml INFα2b for 48 h, relative to untreated control cells. Each value represents mean± SD of three independent experiments. Note: * denotes p <0.05.

To test if the interferon sensitivity of HPV-positive SiHa cells was due to reduced E6 and E7 viral protein levels, we tested the effects of IFNα2b and binase on HPV-negative cervical carcinoma cells C33A, which do not express E6 and E7 proteins. The IC_50_ for binase 7.6±3.1 μM for C33A cells at 72 h (Supplementary Figure 1B), which was lower than that of SiHa cells (Supplementary Figure 1A). The viability of C33A cells was reduced by 59% at 72 h after treatment with 8 μM binase or combination of 8 μM binase plus 100 ng/ml IFNα2b (Figure 4B). Treatment with either binase or a combination of binase and IFNα2b did not enhance apoptosis in C33A cells at 48 h (Figure 6). Binase treatment did not alter p53 levels in C33A cells while IFNα2b increased p53 levels (Figure 7). Moreover, treatment with either binase or a combination of binase and IFNα2b did not alter pRb levels in C33A cells (Figure 7).

## DISCUSSION

Vaccination remains the most effective treatment against HPV by protecting individuals against contracting the virus and preventing its manifestation as warts, cervical ectoption and the prospective development of HPV-associated cancers [1]. However, vaccination is ineffective in HPV infected individuals. In addition, vaccination of the large world population, especially third world countries where HPV-infection is most widespread is challenging. There are no effective means to eliminate HPV from infected individuals and prevent the development of the associated diseases [21]. Current treatment of HPV infections relies mostly on removal of warts and HPV-infected lesions through various ablative and non-surgical therapies. Prospective vaccines based on patient-derived dendritic cells are very expensive, logistically problematic and need to be tailored individually for each patient. Hence, there is a high demand for new, effective, comprehensive and inexpensive preventive and therapeutic avenues against HPV-associated diseases.

Binase is selectively toxic for many cancer cells and inhibits tumor growth and metastasis in animal models [10, 17]. It does not affect normal cells or induce specific T-cell immune responses, but has hepatoprotective properties [18, 22]. Furthermore, binase demonstrates antiviral activities against rabies, influenza and other viruses [11, 19]. We demonstrated that the combined anticancer and antiviral activities of binase are responsible for its selective toxicity against HPV-positive cervical carcinoma SiHa cells, but not against HPV-negative C33A cells (Figures 1, 2 and 6). The efficiency of binase antitumor activity depends on the expression of specific oncogenes, which are downregulated by binase treatment [14]. The malignant transformation state of SiHa cells is maintained by the expression of the high-risk HPV-16 encoded E6 and E7 proteins [23]. In the present study, we demonstrated that binase treatment of SiHa cells resulted in substantial downregulation of the viral E6 and E7 proteins (Figure 3) and increased the levels of tumor suppressor proteins, p53 and pRb, thereby inducing apoptosis and eliminating the infected cells (Figures 2, 4, and 5). The HPV-negative C33A cells that do not express E6 and E7 viral proteins were less sensitive to binase and therefore did not upregulate p53 and pRb following binase treatment. This suggests that the effect of binase on p53 and pRb proteins in SiHa cells is due to downregulation of the HPV proteins, E6 and E7.

In addition to altering expression and function of tumor suppressor proteins, the viral E6 and E7 proteins alter the innate immune response by suppressing type I IFN signaling [6]. The expression of viral oncogenes, particularly E7, is significantly higher in patients that are unresponsive to the IFN treatment than in responsive patients [24]. This suggests that downregulation of E6 and E7 by binase releases the suppression of the IFN pathway, thereby restoring the IFN response. Hence, SiHa cells that show low sensitivity to IFNα2b are substantially sensitized by the combined treatment with IFNα2b and binase (Figures 2, 4, and 5). The HPV-negative C33A cells treated with a combination of IFNα2b and binase show similar sensitivity to C33A cells treated with binase only (Figure 6).

**Figure 8:**
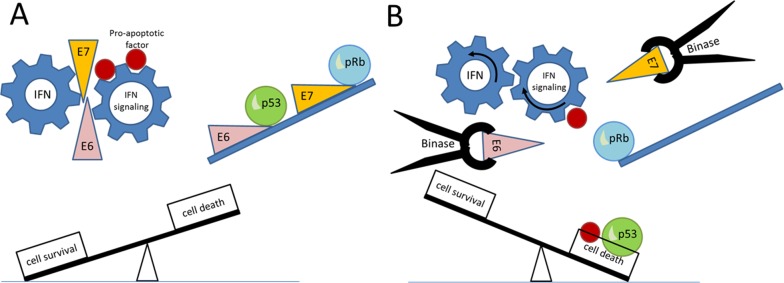
Schematic representation of the effect of binase on HPV-positive cervical carcinoma cells expressing *E6* and *E7* viral oncogenes **(A)** E6 and E7 proteins are shown as pink and orange wedges, which suppress interferon response as well as tumor suppressor proteins, p53 and pRb (green and blue balls). **(B)** Treatment with binase (black tongs) decreases E6 and E7 levels, thereby restoring INF response and p53 and pRb levels, which induce cancer cell death.

Figure 8 shows the probable mechanism by which binase suppresses *E6* and *E7* viral oncogenes in HPV-positive cells. E6 and E7 proteins suppress interferon response and tumor suppressor proteins, p53 and pRb, thereby enhancing cell survival. Binase treatment decreases E6 and E7 levels probably by inhibiting viral replication and *E6*/*E7* expression. This results in restoring IFN signaling and restoration of the p53 and pRb, thereby inducing cell death.

In conclusion, we demonstrated that binase treatment decreases E6 and E7 viral proteins in HPV-16 positive SiHa cells. This results in higher p53 and pRb tumor suppressor protein levels and restores IFN signaling (Figure 8). Therefore, the combined use of binase and IFN would not only effectively eradicate HPV-positive cervical cancer cells, but also prevent malignancy due to HPV-related lesions such as genital warts, cervical intraepithelial neoplasia, as well as post-surgical re-infection with high risk HPVs.

## MATERIALS AND METHODS

### Binase enzyme preparation and purification

The purified 12.3 kDa Binase enzyme was prepared from *Escherichia coli* BL21(DE3) with pGEMEX1/ent/binase plasmid as described previously [25, 26]. Endotoxin contamination in binase preparations was determined by the Limulus amoebocyte lysate test (LAL; Charles River Endosafe) and was less than 5 EU/mg. Binase activity was determined using poly(I) as substrate as described previously [27].

### Cell culture

SiHa and C33A epithelial cell lines were obtained from the American Type Culture Collection (ATCC, Manassas, Virginia, USA). The SiHa and C33A cells were grown in RPMI-1640 and DMEM media, respectively, supplemented with 10% FBS, 2mM L-glutamine, 100 units/mL penicillin and 100 mg/mL streptomycin at 37°C in a humid atmosphere with 5% CO_2_.

### IFNα2b gene cloning and plasmid construction

The *IFNα2b* gene was assembled from several chemically synthesized overlapping oligonucleotides by PCR. Its nucleotide sequence was optimized based on codon usage in highly expressed *E. coli* genes. The *IFNα2b* gene was fused to 3′-terminus of the *SUMO* [28] gene by overlapping PCR with the intermediate amino acid sequence at the junction to generate a fusion protein,(SUMO)-QIGG-(IFNα2b)-CDLP. The 5′-terminus of *SUMO* gene was extended by the amino acid sequence, GHHHHHHGS. The hybrid *SUMO-IFNα2b* gene was cloned into pET39b plasmid vector between restriction sites, *Nde*I and *Hin*dIII.

### IFNα2b isolation and purification

The SUMO-IFNα2b gene was expressed in the *E. coli* strain BL21 (DE3). The BL21 (DE3) cells were grown overnight in the ZYM-5052 autoinduction medium containing 200 μg/ml Kanamycin [29]. Then, the cells were harvested and disrupted by sonication. The insoluble fraction was pelleted at 50,000 xg for 30 min at 4°C, and the supernatant was loaded onto a 1 ml HisTrap HP column (GE Healthcare). After washing, the bound proteins were eluted with a linear gradient of 30 – 500 μM imidazole. The fractions containing SUMO-IFNα2b were pooled and digested at 6°C overnight with SUMO hydrolase (ULP1) in a1:200 molar ratio (enzyme:substrate). Then, the protein solution was desalted and concentrated with Amicon Ultra-15 centrifugal filter and reapplied to a 1 ml HisTrap HP column (GE Healthcare). The flow through was collected and loaded onto Mono Q 5/50 GL column. The bound protein was eluted with a linear 0 – 1 M NaCl gradient. The fractions were analyzed by 15% reduced SDS-PAGE. The fractions containing IFNα2b were pooled and concentrated with Amicon Ultra-15 centrifugal filter and then sterile filtered through a 0.22 μm membrane filter. Endotoxin levels in IFNα2b preparation determined by the Limulus amoebocyte lysate test (LAL; Charles River Endosafe) and were less than 100 EU/mg. The activity determined by the viral resistance test was not less than 2×10^8^ IU/mg and the purity was ≥97% as determined by HPLC (See Supplementary Figure 2).

### Real time cell index analysis

Real time cell index analysis was performed to measure focal adhesion of live cells using xCELLigence real time cell analyser (RTCA; ACEA Biosciences) [30]. The xCELLigence biosensor measured cellular adhesion and the cell index (unitless) was determined by the xCELLigence software (version 1.2.1). Cells were seeded onto custom RTCA E16 plates (ACEA Biosciences), coated with high-density gold arrays for measuring electric impedance. The cells were incubated for 24 h to attain a stable cell index followed by treatment with binase or combination of binase and IFNα2b. Cell index measurements were recorded every 15 min during the course of cell proliferation.

### WST-1 cell viability assay

Cell viability was assessed with a WST-1 test kit (Roche Diagnostics), which is based on the cleavage of water-soluble tetrazolium salt by mitochondrial dehydrogenases in live cells. SiHa cells were seeded in 96-well plates and cultured for 24 h at 37°C. Then, the cells were treated with binase and/or IFNα2b for 48-72 h followed by incubation with WST-1 reagent for 60 min at 37°C. The absorbance of samples was measured in a multiscan FC microplate reader (Thermo Fisher Scientific) at 450 nm. A mixture of cell-free medium with the WST-1 reagent was used as a background control. The activity of mitochondrial dehydrogenases was calculated as the difference in absorbance between each sample and the background control. Respiratory activity of untreated cells was taken as 100%. The experiment was performed in triplicate and reported as mean± SD.

### Flow cytometry analysis of apoptosis

We performed flow cytometry analysis to determine the percent apoptosis/necrosis by double staining with Pacific Blue conjugated Annexin-V (Molecular Probes) and propidium iodide (PI; Sigma). The cells were first washed with PBS at 4°C and resuspended in 0.1 ml (1×10^6^ cells/ml) of buffer-A (10 mM Hepes, 140 mM NaCl, 2.5 mM CaCl_2_, pH 7.4). Then, they were incubated with 5 μl of Pacific Blue-conjugated Annexin V (Ex/Em 405/455 nm) for 15 min at room temperature in darkness. Then, 400 μl of buffer-A was added and incubated with 10 μg/ml PI (Ex/Em 493/632 nm) for 1–2 min before analysis in a GALLIOS flow cytometer (Beckman Coulter). The typical forward and side scatter plot showing distribution of SiHa and C33a cells by size and granularity is shown in the Supplementary Figure 3A, 3B. In our analysis, we excluded cell debris lying outside the R1 gate (Supplementary Figure 3). We analyzed atleast10000 cells for each sample by Annexin-V Pacific Blue (FL9) versus propidium iodide (FL4) (Supplementary Figure 3B). The Annexin-V^+^ PI^−^ cells were considered apoptotic (lower right quadrant), Annexin-V^−^ PI^+^ (upper left quadrant) were considered as necrotic cells, while all Annexin-V^+^ PI^+^ (upper right quadrant), Annexin-V^+^ PI^−^ (lower right quadrant) and Annexin-V^−^ PI^+^ were considered as dying cells (Supplementary Figure 3B). The apoptotic and dying cells were expressed as a percentage of the total number of cells. The experiments were repeated thrice and were expressed as mean±SD.

### Western blotting

The harvested cells were washed with ice-cold PBS and solubilized in ice-cold lysis buffer (25 mM Tris-HCl, pH 7.6, 150 mM NaCl, 1% Nonidet-P40, 0.1% SDS, 1% sodium deoxycholate, 1 μM of PMSF) with constant stirring at 4°C for 1 h. The cell lysates were centrifuged at 13000 xg for 10 min and the protein supernatants were collected and quantified. Equal amounts of protein lysates were separated on SDS-PAGE and transferred onto PVDF membranes. The membranes with separated proteins were blocked with 5% skimmed milk in 1X PBST for 1 h. Then, the membranes were incubated overnight at 4°C with the following primary antibodies: HPV16 E6 (ab70, dilution 1:500) and HPV16 E7 (ab30731, dilution 1:1000) from Abcam; p53 (sc-126, dilution 1:1000) from Santa Cruz Biotechnology; pRb (9309s, dilution 1:2000) from Cell Signaling; β-actin (AM4302, dilution 1:15000) from Ambion. Then, the blots were incubated with the appropriate horseradish peroxidase-conjugated secondary antibodies and developed by the enhanced chemiluminescence SuperSignal™ West Femto Maximum Sensitivity Substrate kit (Thermo Scientific). Chemiluminescence was detected using Bio-Rad ChemiDoc MP instrument and the protein bands were analyzed by densitometry using the Image Lab program (Bio-Rad).

### Statistical analysis

The data are shown as mean ± standard deviation measure from triplicate values obtained from 3 independent experiments. The differences among the groups were analyzed by Student's t-test and *p* < 0.05 was considered statistically significant. Statistica 7 software was used for analysis.

## SUPPLEMENTARY FIGURES



## Abbreviations

HPV: human papilloma virus
pRb: retinoblastoma protein
IFN: interferon
RNases: ribonucleases
