# Supplementary material for: Binase treatment increases interferon sensitivity and apoptosis in SiHa cervical carcinoma cells by downregulating E6 and E7 human papilloma virus oncoproteins
Source: Oncotarget. 2017 Aug 10;8(42):72666–75. doi: 10.18632/oncotarget.20199 (PMC5641160; doi:10.18632/oncotarget.20199)
Supplement: Supplementary file 1 [file oncotarget-08-72666-s001.pdf]

Binase treatment increases interferon sensitivity and apoptosis in SiHa cervical carcinoma cells by downregulating E6 and E7 human papilloma virus oncoproteins

SUPPLEMENTARY MATERIALS

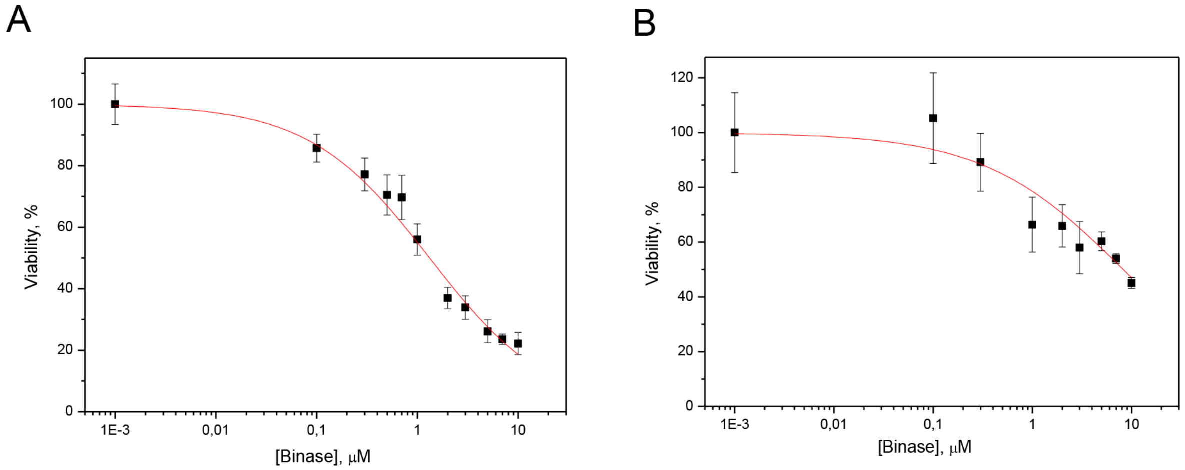

Supplementary Figure 1: Effect of binase on the viability of **(A)** SiHa and **(B)** C33A cells. Cell viability was measured by WST-1 test kit, 72 h after incubation with various concentrations of binase. Each value represents mean± SD of at least three independent experiments with triplicate samples. Values are expressed as a percentage relative to viability of the control without binase treatment.

Time (min)	Mobile phase A	Mobile phase B
0-1	72	28
1-5	67	33
5-20	63	37
20-30	57	43
30-33	40	60
33-37	40	60
37-39	72	28
39-60	72	28

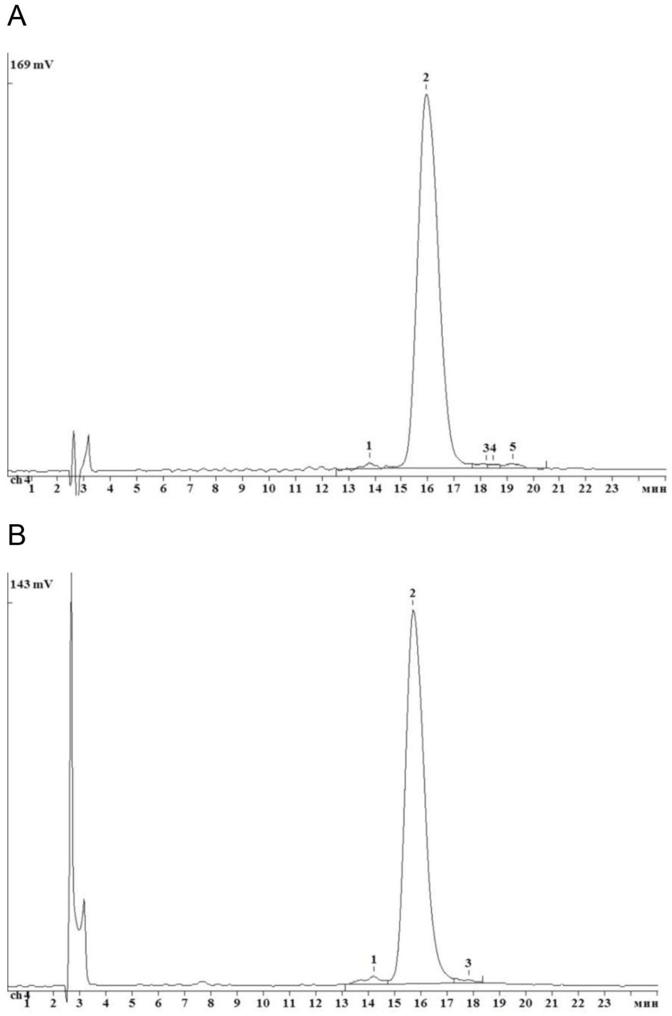

Supplementary Figure 2: Chromatogram profile of IFNα2b preparation. Representative chromatogram profile of **(A)** IFNα2b preparation and **(B)** Reference IFNα2b (WHO standard, NIBSC95/566). Chromatographic separation conditions: column length 250 mm; column diameter 4 mm; column packed with Diaspher 300 C18 (6 µm); temperature: 45ºC; detection at 220 nm; flow rate 1 ml/min; mobile phase A: 30% acetonitrile, 0.2% TFA in water, mobile phase B: 80% acetonitrile, 0.2% TFA in water.

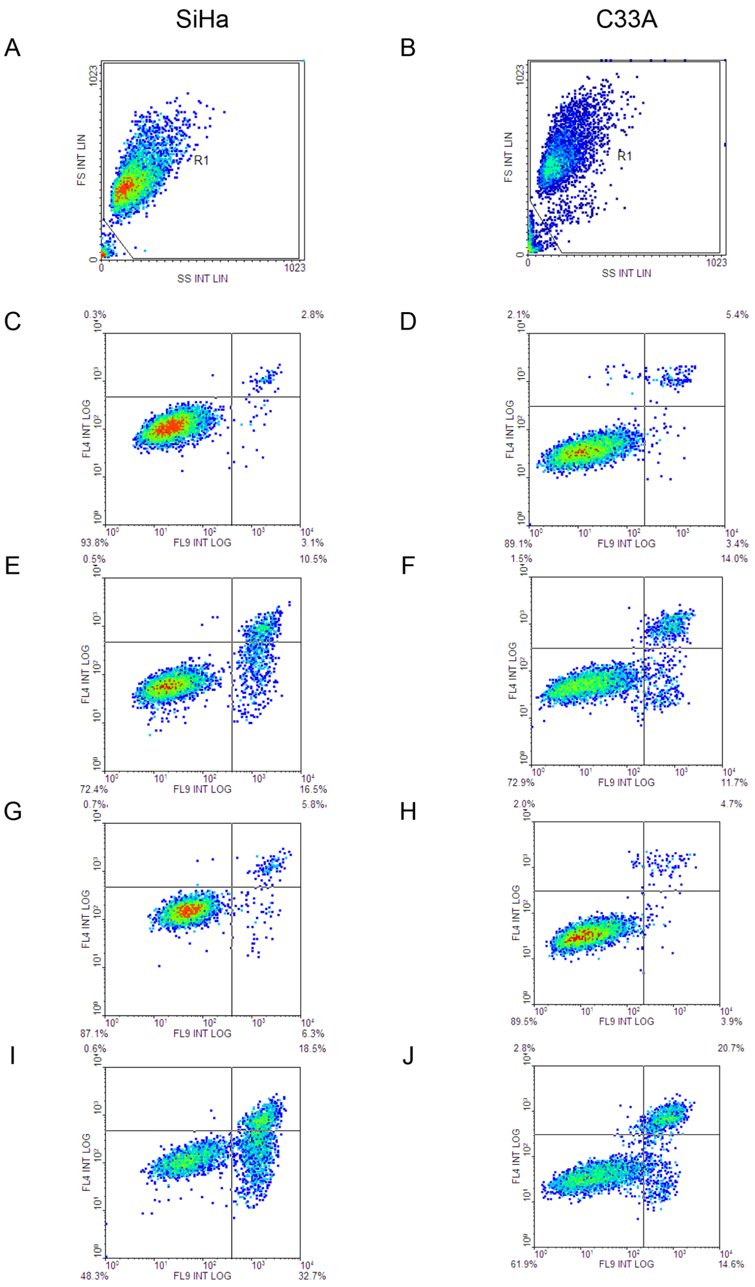

Supplementary Figure 3: Flow cytometry analysis of cytotoxic effects of binase (8 µM) and IFNα2b (100 ng/ml) treatments on SiHa and C33A cells at 48 h. **(A,B)** Flow cytometry density plot showing forward (FS) and side (SS) scatter of (A) SiHa and (B) C33A cells, describing their size and granularity. Cell population is marked by R1 gate. **(C-J)** Flow cytometry density plots showing Annexin-V Pacific Blue (FL9) versus propidium iodide (PI) (FL4) staining in (C,E,G,I) SiHa and (D,F,H,J) C33A cells. Lower left quadrant – AnnexinV$^-$PI$^-$ cells; lower right quadrant - AnnexinV$^+$ PI$^-$ cells; upper right quadrant – AnnexinV$^+$ PI$^+$ cells; upper left quadrant – AnnexinV$^-$ PI$^+$ cells. (C,D) Control cells; (E,F) cells treated by binase; (G,H) cells treated by IFNα2b and (I,J) cells treated by binase + IFNα2b.
